# A Cross-Sectional Study of Dairy Cattle Metagenomes Reveals Increased Antimicrobial Resistance in Animals Farmed in a Heavy Metal Contaminated Environment

**DOI:** 10.3389/fmicb.2020.590325

**Published:** 2020-11-16

**Authors:** Natalia Carrillo Gaeta, Emily Bean, Asha Marie Miles, Daniel Ubriaco Oliveira Gonçalves de Carvalho, Mario Augusto Reyes Alemán, Jeferson Silva Carvalho, Lilian Gregory, Erika Ganda

**Affiliations:** ^1^Department of Internal Medicine, School of Veterinary Medicine and Animal Science, University of São Paulo, São Paulo, Brazil; ^2^Department of Animal Science, College of Agricultural Sciences, Pennsylvania State University, State College, PA, United States; ^3^Intercollege Graduate Degree Program in Integrative and Biomedical Physiology, Pennsylvania State University, State College, PA, United States

**Keywords:** shotgun sequencing, metagenomics, microbiome, heavy metals, antimicrobial resistance, dairy cattle

## Abstract

The use of heavy metals in economic and social development can create an accumulation of toxic waste in the environment. High concentrations of heavy metals can damage human and animal health, lead to the development of antibiotic resistance, and possibly change in bovine microbiota. It is important to investigate the influence of heavy metals in food systems to determine potential harmful effects environmental heavy metal contamination on human health. Because of a mining dam rupture, 43 million cubic meters of iron ore waste flowed into the Doce river basin surrounding Mariana City, Brazil, in 2015. Following this environmental disaster, we investigated the consequences of long-term exposure to contaminated drinking water on the microbiome and resistome of dairy cattle. We identified bacterial antimicrobial resistance (AMR) genes in the feces, rumen fluid, and nasopharynx of 16 dairy cattle 4 years after the environmental disaster. Cattle had been continuously exposed to heavy metal contaminated water until sample collection (A) and compared them to analogous samples from 16 dairy cattle in an unaffected farm, 356 km away (B). The microbiome and resistome of farm A and farm B differed in many aspects. The distribution of genes present in the cattle’s nasopharynx, rumen, and feces conferring AMR was highly heterogeneous, and most genes were present in only a few samples. The relative abundance and prevalence (presence/absence) of AMR genes were higher in farm A than in farm B. Samples from farm A had a higher prevalence (presence) of genes conferring resistance to multiple drugs, metals, biocides, and multi-compound resistance. Fecal samples had a higher relative abundance of AMR genes, followed by rumen fluid samples, and the nasopharynx had the lowest relative abundance of AMR genes detected. Metagenome functional annotation suggested that selective pressures of heavy metal exposure potentially skewed pathway diversity toward fewer, more specialized functions. This is the first study that evaluates the consequences of a Brazilian environmental accident with mining ore dam failure in the microbiome of dairy cows. Our findings suggest that the long-term persistence of heavy metals in the environment may result in differences in the microbiota and enrichment of antimicrobial-resistant bacteria. Our results also suggest that AMR genes are most readily detected in fecal samples compared to rumen and nasopharyngeal samples which had relatively lower bacterial read counts. Since heavy metal contamination has an effect on the animal microbiome, environmental management is warranted to protect the food system from hazardous consequences.

## Introduction

Despite the substantial advancements brought by industrialization, it is well known that incorrect management of industrial activity has the ability to cause severe damage to environmental, human, and animal health. Heavy metals are bio-accumulative elements that naturally occur in water and soil (Guilherme et al., 2005), but human activities such as mining can result in the environmental accumulation of heavy metals as toxic waste. Heavy metal contamination is a public health concern due to the presence of these toxic elements in food crops (Rai et al., 2019), milk (Zhou et al., 2019) and meat (Wang et al., 2019). Chronic heavy metal intake of foods derived from plants grown or animals raised in a contaminated environment can lead to bio-accumulation in the organism and multiple health risks (Jaishankar et al., 2014). Gastrointestinal cancer (Yuan et al., 2016), fetal growth restriction (Sabra et al., 2017), and neurological diseases (Chen et al., 2016; Karri et al., 2016; Bjorklund et al., 2018) are examples of heavy metal-related consequences in humans.

Increased heavy metal concentration in the environment has been linked to increased prevalence of antibiotic resistance in bacteria by a co-resistance phenomenon (Nies, 1992; Silver, 1996; Wireman et al., 1997; Choudhury and Srivastava, 2001; Baker-Austin et al., 2006). Co-resistance is characterized by the closeness between different types of resistance genes located in the same genetic element, such as plasmids and transposons, which are often transferred together. As a result of this connection, the transfer of one gene (heavy metal resistance) may occur in concert with the transfer of the closest gene (antibiotic resistance) (Baker-Austin et al., 2006). Consequently, some resistance mechanisms are shared between antibiotics and heavy metals (Silver and Phung, 1996; Levy, 2002; Mukhopadhyay and Rosen, 2002; Roberts, 2005). Efflux pumps are another important resistance system in both gram-negative and gram-positive bacteria. These transmembrane proteins are responsible for pumping antimicrobial (molecules/compounds/substances) (e.g., antibiotics, heavy metals, biocides) out of the cell, regulating their concentrations in the bacterial internal environment (Blanco et al., 2016). Multi-drug resistance efflux pumps are important examples of cross-resistance determinants, and the resistance-nodulation-division (RND) superfamily is of major clinical significance in gram-negative that transports heavy metals, and hydrophobic drugs (Zgurskaya and Nikaido, 2000).

Chronic heavy metal intake may lead to health complications due to its bio-accumulative effect (Jaishankar et al., 2014). Heavy metals are absorbed in the gastrointestinal tract but some also remain in the intestinal lumen, potentially interfering in the microenvironment and its functioning (Breton et al., 2013). For example, rodent models have shown that exposure to lead and cadmium changed the gut microbiota of rats; specifically, the relative abundance of *Lachnospiraceae* decreased and *Lactobacillaceae* and *Erysipelotrichaceacae* increased, resulting in gut dysbiosis associated with colitis and gut inflammation (Lepage et al., 2011; Breton et al., 2013). Bacteria unique to ruminants have been shown to be sensitive to heavy metals *in vitro*. An *in vitro* study of rumen environments showed that *Bacteroides succinogenes*, *Ruminococcus albus*, *Bacteroides amylophilus*, and *Eubacterium ruminantium* were sensitive to heavy metals, such as cadmium, mercury, copper, arsenic, and chromium (Forsberg, 1978). It is well-established that the rumen resident microorganisms are responsible for feed fermentation and production of important metabolites that maintain ruminant homeostasis; thus, it is important to evaluate the impact of heavy metal exposure on ruminal microbiome.

Anthropogenic activity (e.g., mining) is responsible for the deposition of heavy metals in the environment (Nguyen et al., 2019). In 2015, the rupture of a Brazilian mining dam resulted in iron ore waste flowing into the Doce river basin, which resulted in more than 43 million cubic meters of iron ore waste flowing into the Doce river basin surrounding Mariana City in the state of Minas Gerais (Samarco, 2016; do Carmo et al., 2017). Analysis of Doce river water 2 years after the event revealed aluminum, nickel, arsenic, copper, manganese, and lead higher than the maximum value allowed by Brazilian legislation (de Carvalho et al., 2017). Little is known about the effects of heavy metal environmental contamination on food production animals and their metagenomes. This presented the opportunity to investigate the consequences of long-term exposure to heavy metal contaminated drinking water on the dairy cattle metagenome. Thus, we performed a cross-sectional study to compare the prevalence and scope of antimicrobial resistance (AMR) genes between a contaminated farm and an unaffected farm 356 km away, in addition to characterizing the impact of prolonged exposure on the nasopharynx, rumen fluid, and fecal microbiomes.

## Materials and Methods

This research was conducted from August 2018 through November 2019 (sample collection in farm A and farm B was performed in August 2018 and October 2018, respectively). Sequencing and analysis of the metagenome were performed from August to November 2019. Sixteen dairy cows from a potentially heavy metal contaminated environment (farm A) and 16 dairy cows from a non-contaminated environment (farm B) were sampled at three body sites. The research protocol was reviewed and approved by the Ethics Committee in the Use of Animals of the School of Veterinary Medicine and Animal Science of the University of São Paulo (protocol no. 6424070217).

### Animals and Facilities

A convenience sample of 32 dairy cows was obtained from two different farms located in Minas Gerais within 526 km of each other and were enrolled in this study.

Sixteen dairy cows were selected from a dairy farm located in Mariana City (Latitude: 20° 22′ 41″ S, Longitude: 43° 25′ 0″ W), Minas Gerais State, Brazil (farm A) in a potentially heavy metal contaminated area (de Carvalho et al., 2017). Cows were 3 years old or older to be enrolled in the study, ensuring that they were present at the farm during the environmental accident. Cows were housed in a single pen, grazed on pastures, and drank the affected river water. In addition to pasture, cows received mineral salt, wheat bran, and cornmeal. All animals were vaccinated for rabies and foot and mouth disease.

Sixteen cows were selected from a dairy farm located in Caldas City (Latitude: 21° 55′ 23″ S, Longitude: 46° 23′ 15″ W), Minas Gerais State, Brazil (farm B). The cows grazed on pastures and drank water from a non-contaminated lagoon. In addition to pasture, cows received corn and soybean meal as well as mineral salt. All animals were vaccinated for leptospirosis and foot and mouth disease.

Samples were collected from females on both farms. Both owners referred to cases of mastitis and reproductive disorders during the year before sampling time. The cows received antibiotic treatment on the farm when necessary. Unfortunately, health records were not available for every animal.

### Data Collection

Two veterinarians from our research group performed physical examinations on each cow, assessing vital parameters (respiratory and heart rates, rectal temperature, and ruminal movements) and any clinical sign that indicated sickness. Animals displaying any symptoms of illness were removed from the study. Decreased milk production was also reported by the farmer for animals in the affected farm.

### Sample Collection

Samples were collected from the nasopharynx, rumen, and rectum. Deep nasopharyngeal swabs (DNS) were collected using an 80 cm sterile swab (Provar, Brazil) covered by a thin sterile plastic sheath. The sample was obtained from the left nostril only. Mucus and dirt were removed from the nostrils with a paper towel, followed by inserting the swab into the nasopharyngeal cavity until met with resistance. The swab was passed 10 times over the respiratory mucosa before being removed from the nostril. The swabs were placed in cryogenic tubes and immediately stored in liquid nitrogen. Fecal samples were obtained by introducing a sterile swab into the rectum and rotating it for 15 s. Each swab was immediately stored in liquid nitrogen in a cryogenic tube. Five milliliters of ruminal fluid was obtained by introducing a plastic probe into the mouth until it reached the rumen. Using a vacuum system, the content was collected in a plastic bottle and immediately stored in liquid nitrogen. The system was cleaned with water and 70% alcohol between animals.

### DNA Extraction

Before DNA extraction, all swab samples were pre-processed to obtain the maximum DNA concentration. Briefly, one milliliter of sterile PBS buffer 1× (pH: 7.4) was added to all swab samples and vortexed for 30 min. The swabs were removed, and samples were centrifuged at 3,000 × *g* for 30 min. The supernatant was discarded, and the pellet was resuspended in 300 μl of PBS. Rumen fluid samples were thawed and vortexed before the extraction. DNA extraction was performed using the MagMAX^TM^ CORE Nucleic Acid Purification Kit (Thermo Fisher Scientific Inc., Waltham, MA, United States), according to manufacturer instructions using 200 μl of starting sample (concentrated swab material or rumen fluid). The DNA concentration was measured by a spectrophotometer (Nanodrop, Thermo Fisher Scientific Inc., Waltham, MA, United States).

### Metagenome

Before the library preparation, the DNA concentration of each sample was also assessed by Qubit Fluorometric Quantification (Thermo Fisher Scientific, San Jose, CA, United States). Ten nanograms of each Qubit quantified genomic DNA was sheared with a Covaris E220 instrument operating SonoLab v6.2.6 generating approximately 300 bp DNA fragments according to the manufacturer’s protocol. Between 10 and 100 ng of fragmented DNA was processed into Illumina compatible sequencing libraries using sparQ DNA Library Prep Kit (Quantabio, Beverly, MA, United States). Each library was barcoded with unique dual index sequences (NEXTFLEX^®^ Unique Dual Index Barcodes, BioO Scientific). Library size and amount were confirmed with a Bioanalyzer High Sensitivity DNA chip. Polymerase chain reaction primers and reagents included in the sparQ kit were used to perform PCR, and products were purified with AMPure XP beads. Equimolar libraries were pooled and subjected to Illumina NovaSeq 6000 sequencing at 2 × 150 bp (Illumina, San Diego, CA, United States). Shotgun whole metagenome sequencing was performed at the Genome Sciences and Bioinformatics Core at the Pennsylvania State University College of Medicine, Hershey, PA, United States. Illumina bcl2fastq (released version 2.20.0.422) was used to extract de-multiplexed sequencing reads.

### Data Analysis

#### Microbiome

Fastq files were uploaded to the MG-RAST server (Keegan et al., 2016), concatenated and submitted to their standard pipeline to determine the relative abundance of the microbiota. In the MG-RAST pipeline, sequences were subject to quality control, including dereplication, removal of host-specific species sequences (*Bos taurus*, UMD v3.0), ambiguous base filtering, and read length filtering. Fragments were mapped against a non-redundant database (N5nr). Organism abundance was analyzed with a maximum *e*-value of 1 × 10^–5^, minimum identity cutoff of 60%, and a minimum alignment length cut-off of 15 (default values).

The normality of the data was assessed by the Shapiro–Wilk test (Shapiro and Wilk, 1965). Variances were analyzed by Bartlett’s test (Bartlett, 1937). Relative abundance was calculated by dividing the number of reads of each taxa by the total number of reads of all taxa. Relative abundances of each phylogenetic level (phylum and genus) were analyzed by the Wilcoxon rank-sum test (Wilcoxon, 1945) according to herd (farm A and farm B). Results were described as mean (M) and standard error of the mean (SEM). Differential abundance analysis was performed at the genus level to compare farm A and farm B within each anatomical site with the DESeq2 package in R. The analysis was performed with Wald significance test and mean dispersal type, and *P* values were adjusted with False Discovery Rate. All statistical analyses were completed in R software version 3.6.0 (RStudio Team, 2020). Significance was accepted at *P* < 0.05.

#### Resistome

**FIGURE 1:**
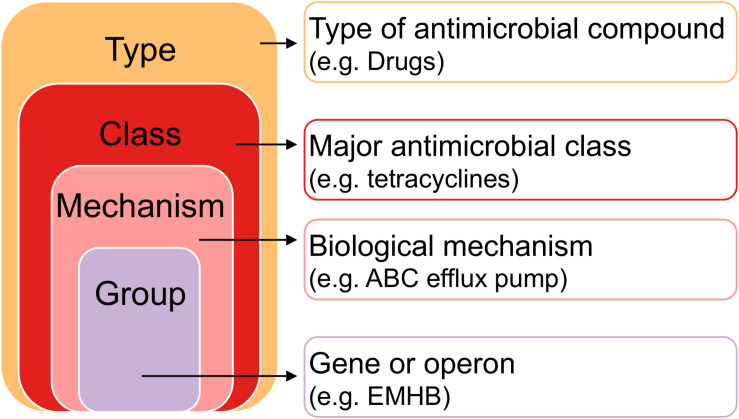
Hierarchy of antimicrobial resistance genes. Each gene or operon (“Group” level) belongs to a Mechanism, a Class, and a Type. Pairwise comparisons between farms and anatomical sites were made between Types, Classes, Mechanisms, and Groups to determine resistome differences.

Read quality was visualized on a random subset of 10 samples with FastQC (Andrews, 2010). Reads were trimmed with Trimmomatic for a minimum length of 50 bp (Bolger et al., 2014). Paired-end reads were merged in FLASH (Magoc and Salzberg, 2011) and converted to FASTA format in Seqkit (Shen et al., 2016). Reads were aligned to a custom AMR gene database built in BLAST (Altschul et al., 1990). The BLAST database was created from the publicly available MEGARes 2.0 AMR database^1^, which incorporates genes from CARD, ARGANNOT, AMRFinder, NCBI, ResFinder, BacMet, Lahey, and ResFinder for a total of 7,868 genes or operons that confer metal, biocide, drug, or multi-level resistance (Doster et al., 2019). The BLAST alignment resulted in a tabular list of reads that aligned to resistance genes or operons, hereon referred to as “gene counts” (Figure 1). Contaminant genes were detected and removed with the decontam R package based on negative control samples from DNA extraction (*n* = 2) and PCR (*n* = 1) (Davis et al., 2017). A mock community sample was used as a positive control at both DNA extraction and PCR, and both underwent sequencing for quality control purposes (Zymo Research, United States).

To account for bias, gene counts were normalized both to gene length and pseudo-sequencing depth. Gene lengths varied for the different mutations found in each gene, so gene counts were divided by the minimum gene length and then the number of merged reads per sample. The normalized data was scaled to relative abundance where the sample with the highest normalized gene count was set to 1. After scaling to relative abundance, we realized the data was strongly zero skewered and the distribution was not corrected by variable transformation. Therefore, we also analyzed the data in terms of gene presence, where data was converted to binary presence per sample (1 if the gene was present in the sample, 0 if not) as this represented the heterogeneity of the data. This is called “gene prevalence” throughout. Higher gene prevalence is defined as higher per-sample average gene presence; total gene presence per farm divided by the number of samples in each farm.

Non-parametric tests were used for pairwise comparisons of relative gene abundance. Significance thresholds were set at a False Discovery Rate corrected *P* < 0.05 (Benjamini and Hochberg, 1995). Separate comparisons were made for resistance type, class, mechanism, and group (Figure 1). Comparisons of relative gene abundance between farms were made with the Wilcoxon Rank-Sum test with False Discovery Rate adjustment for multiple comparisons. Comparisons of relative gene abundance among anatomical sites at each farm were made using the Kruskal–Wallis test with False Discovery Rate adjustment for multiple comparisons.

#### Metagenome Functional Annotation

To determine whether farm A was enriched in functions that promote resistance to both antimicrobials and heavy metals, we utilized *mi*-faser, a high-precision metagenome analysis pipeline which maps sequencing reads to molecular functions encoded by the read-corresponding genes, to annotate our data as a set of molecular functions (Zhu et al., 2018). Paired-end fastq files were uploaded to the *mi*-faser submission site^2^ whose pipeline performed quality control with fastp (phredquality = 20, readlength = 40) before reads were mapped to the *mi*-faser extended reference database. Job output files including enzyme catalog (E.C.) numbers, functional annotation, and read counts per annotation were exported as .csv for all 90 samples. A Wilcoxon Rank-Sum test was performed to compare the number of unique reads identified between farms by sampling sites. Reads were then normalized according to the total read count in each individual metagenome and these data together with their metadata (Sample ID, farm, sampling site) were imported into the open-source machine learning software Waikato Environment for Knowledge Analysis (WEKA) (Frank et al., 2016). Within WEKA, the “InfoGainAttributeEval” attribute evaluator algorithm and search method “Ranker” were used to calculate the relative worth of each E.C. by measuring the information gain with respect to three nominal classes (1) “farm” (two levels), (2) “sampling site” (three levels), and (3) a composite class of “farm by site” (six levels). The data set was reduced by selecting the top 10 ranked E.C.s from each approach for pathway mapping in the Kyoto Encyclopedia of Genes and Genomes (KEGG) Mapper (Kanehisa and Sato, 2020).

All statistical analyses were completed in R software version 3.6.0 (RStudio Team, 2020). Code and raw data are available at https://github.com/gandalab/amr-heavymetal.

## Results

Thirty-two dairy cows were selected from two dairy farms, one of which had heavy-metal contaminated drinking water. At the time of sampling, no clinical manifestations that indicated any disease was observed in any animal. Farm A’s owner reported to decreased in milk production after the dam failure (250 L milk/per to 200 L milk/per day). No differences were noted by farm B’s owner.

Rumen fluid, fecal swabs, and DNS were collected from each cow, totaling 96 samples. Six samples from farm B (one fecal swab, three rumen fluids, and two DNS) did not yield enough DNA concentration and were not considered for sequencing, so a total of 90 samples were sequenced and analyzed, in addition to appropriate negative and positive controls. Detailed sequencing data on total number of sequences and base pairs, qualified reads per sample and average length of sequences are presented in Table 1.

**TABLE 1:** Descriptive data on total sequences and base pairs, high quality sequences and average length sequences.

**Features**	**Fecal Swab**	**Rumen Fluid**	**Nasal Swab**
Total sequences	1,618,872,761	1,623,606,104	1,665,839,299
Total base pairs	305,622,131,406	289,694,665,004	313,199,561,929
High quality sequences	40,202,077	45,029,282	1,550,130
Average length (bp)	182	178	174

### Microbiome

**FIGURE 2:**
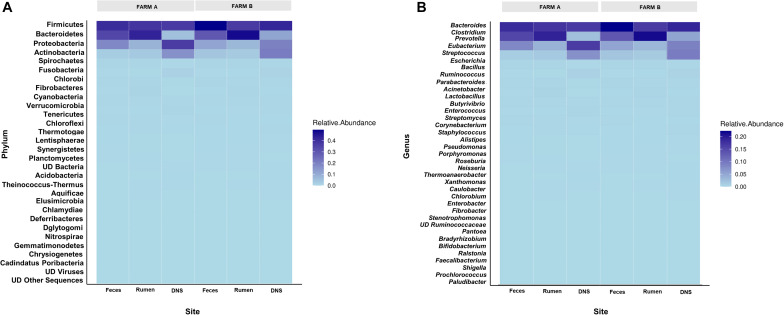
Relative abundance of phyla and genera across farms and sites. Heat maps showing the relative abundance of most abundant phyla **(A)** and genera **(B)** determined on farm A and farm B in fecal swab, rumen fluid and deep nasopharyngeal swab of dairy cattle.

**FIGURE 3:**
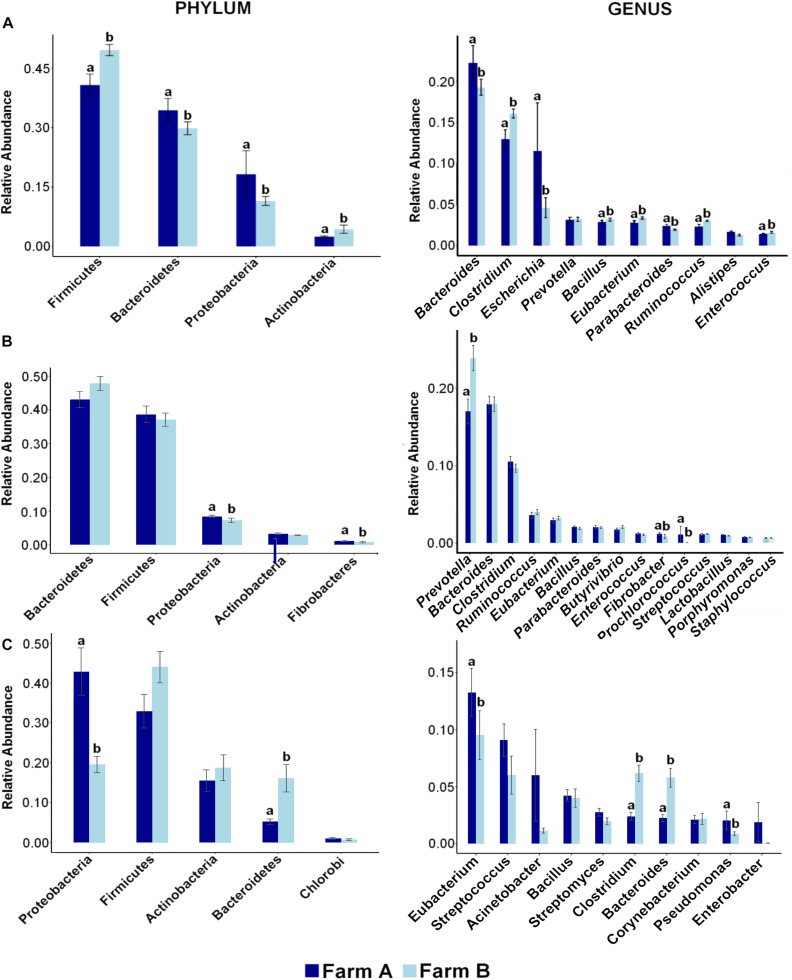
Distribution of most abundant phyla and genera reveals differences across farms in each site. Bar plots showing the relative abundance of most abundant phyla (left) and genera (right) and differences between farm A (dark blue) and farm B (light blue) in **(A)** fecal swab, **(B)** rumen fluid, and **(C)** deep nasopharyngeal swab of dairy cattle. Pairwise comparisons were made between farms with Wilcox Sum test and different letters denote differences between farms (*P* < 0.05) within each taxa.

Twenty-eight phyla were detected in fecal samples (Figure 2A). Firmicutes, Bacteroidetes, and Proteobacteria were the most abundant phyla in all fecal samples (Supplementary Figure S1). Firmicutes (A: 0.40 ± 0.02; B: 0.49 ± 0.01) and Actinobacteria (A: 0.02 ± 0.00; B: 0.04 ± 0.01) were more abundant in farm B than farm A (*P* < 0.05). Bacteroidetes (A: 0.34 ± 0.02; B: 0.29 ± 0.01), and Proteobacteria (A: 0.18 ± 0.05; B: 0.11 ± 0.01) were more abundant in farm A (*P* < 0.05) (Figure 3A). Analysis at the genus level revealed that fecal samples were dominated by 20 genera (Figure 2B). *Bacteroides*, *Escherichia*, and *Clostridium* were the most abundant genera in all fecal samples (Supplementary Figure S2). *Clostridium*, *Bacillus*, *Eubacterium*, *Ruminococcus*, and *Enterococcus* were higher in farm B (*P* < 0.05). *Bacteroides* (A: 0.22 ± 0.02; B: 0.19 ± 0.00), *Escherichia* (A: 0.11 ± 0.05; B: 0.04 ± 0.01) and *Parabacteroides* (A: 0.02 ± 0.00; B: 0.01 ± 0.00) were more abundant in farm A (*P* < 0.05) (Figure 3A). Differential abundance analysis revealed that 220 fecal genera of 600 total differed between farms. The 25 most abundant genera in farm A compared to farm B and the 25 most abundant in farm B compared to farm A are shown in Supplementary Table S1.

Phylum analysis of rumen fluid revealed 28 phyla (Figure 2A), in which Bacteroidetes and Firmicutes were the most abundant phyla in all rumen fluid samples (Supplementary Figure S1). The relative abundance of Bacteroidetes and Firmicutes was similar in both farms (*P* > 0.05) (Figure 3B). *Proteobacteria* was more abundant in farm A (A: 0.08 ± 0.00; B: 0.07 ± 0.00) (*P* = 0.02). At the genus level, samples were dominated by 20 genera (Figure 2B), in which *Bacteroides*, *Prevotella*, and *Clostridium* were the most abundant genera in all rumen fluid samples (Supplementary Figure S2). *Prevotella* was more abundant in farm B (A: 0.16 ± 0.01; B: 0.23 ± 0.01) (*P* = 0.01). *Fibrobacter* (A: 0.01 ± 0.00; B: 0.00 ± 0.00) and *Prochlorococcus* (A: 0.01 ± 0.01; B: 0.00 ± 0.00) were more abundant in farm A (*P* < 0.05) (Figure 3B). Differential abundance analysis of 600 bacterial genera in ruminal fluid showed that 92 differed in relative abundance between farms (Supplementary Table S2).

The phylum analysis of DNS revealed 28 phyla (Figure 2A). Proteobacteria, Firmicutes, Actinobacteria, and Bacteroidetes were the most abundant phyla in all DNS samples (Supplementary Figure S1). The relative abundance of Proteobacteria was higher in farm A (A: 0.36 ± 0.05; B: 0.20 ± 0.01) (*P* = 0.001) and Bacteroidetes in farm B (A: 0.05 ± 0.00; B: 0.12 ± 0.03) (*P* = 0.0001) (Figure 3C). Analysis by genus-level revealed samples dominated by 10 genera (Figure 2B), particularly *Eubacterium*, *Streptococcus*, and *Bacillus*, which were the most abundant genera in all DNS samples (Supplementary Figure S2). The genera *Eubacterium* (A: 0.13 ± 0.02; B: 0.001 ± 0.00), *Pseudomonas* (A: 0.02 ± 0.00; B: 0.00 ± 0.00), *Clostridium* (A: 0.02 ± 0.00; B: 0.00 ± 0.00), and *Bacteroides* (A: 0.02 ± 0.00; B: 0.00 ± 0.00) were more abundant in farm A compared to farm B (*P* < 0.05) (Figure 3C). Differential abundance analysis of 594 bacterial genera detected in DNS showed that 199 differed in relative abundance between farms (Supplementary Table S3).

### Resistome

A total of 549 AMR genes (called “groups” by the MEGARes database as it can include either genes or operons; called “gene” throughout) were analyzed after removing contaminant gene alignments (*n* = 74) and genes found only in one sample (*n* = 117). The distribution of AMR gene presence was highly heterogeneous between samples, as most of the 549 genes were found in only a few samples (Supplementary Figure S3).

**FIGURE 4:**
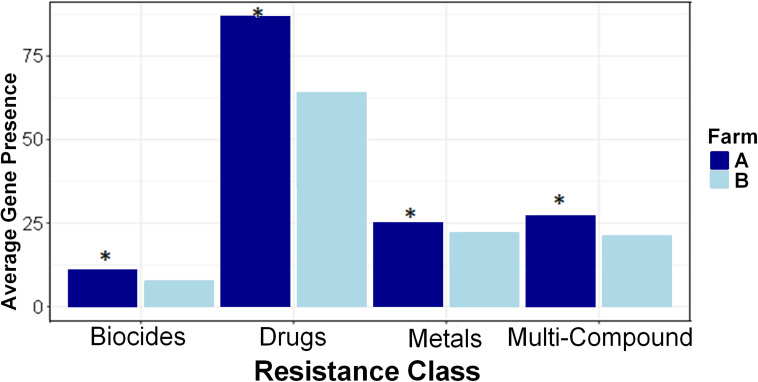
Resistance classes are more prevalent in Farm A than Farm B. Average (per-sample) gene presence is shown within each farm across four resistance classes. Pairwise comparisons were made between farms with Kruskal–Wallis and asterisks denote differences between farms (*P* < 0.05) within each class.

Farm A had a higher relative abundance and higher resistance prevalence in all four AMR classes of biocides, drugs, metals, and multi-compound than farm B (Figure 4). Higher prevalence of biocide resistance in farm A was driven by multi-biocide resistance ABC (normalized per-sample average gene presence 1.375 farm A and 0.628 farm B) and RND (normalized per-sample average gene presence 3.5 farm A and 1.581 farm B) efflux pumps. Metal resistance prevalence was driven by tellurium resistance and multi-metal resistance, notably a multi-metal resistance RND efflux pump (per-sample average gene presence 0.438 farm A and 0.047 farm B). Significant multi-compound resistance genes that were denoted as multi-compound resistance for drugs and biocides included two RND efflux pumps and one Major Facilitator (MFS) efflux pump, which were almost absent from farm B samples; RND efflux pump “MEXY” was present in only 1 sample in farm B, while RND efflux pump “MTRD” and the MFS efflux pump “KDEA” were completely absent from farm B. Several types of drug resistance were more abundant in farm A than farm B (Supplementary Figure S4). Mechanisms conferring resistance to aminoglycosides, beta-lactams, glycopeptides, macrolide-lincosamide-streptogramin B (MLS), mupirocin, mycobacterium, nucleosides, tetracyclines, and multi-drug resistance were all more highly abundant and prevalent in farm A than farm B.

**FIGURE 5:**
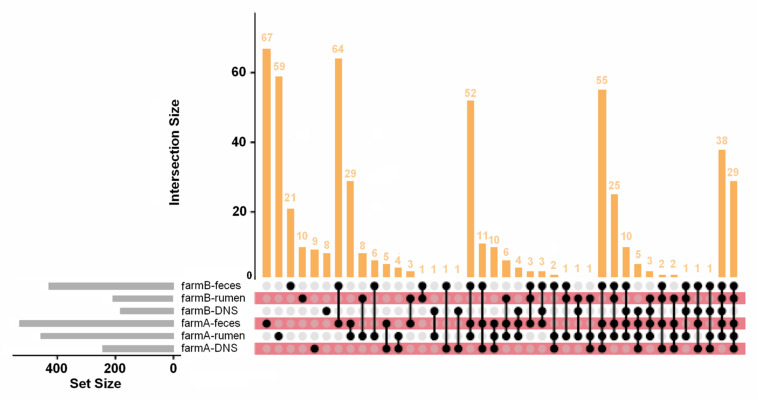
AMR gene distribution across farms and anatomical sites. UpSet plot of AMR gene distribution in each farm and anatomical site. Of the 549 AMR genes detected, 67 are found only in farm A fecal samples and 59 are only in farm A rumen fluid samples. The intersections of gene combinations are shown in the dot matrix and vertical bar plot. The set size (number of genes in each set) is shown in the horizontal gray bar plot. Most genes were detected in fecal and rumen fluid samples compared to DNS samples.

Within each farm, there were strong differences in AMR abundance and prevalence among anatomical body sites and the differences were consistent between farms. Generally, in both farms fecal samples had higher gene presence than rumen fluid samples, and DNS had the least amount of gene presence (Figure 5). Resistance types of biocides, drugs, metals, and multi-compound resistance differed between body sites on both farms. Interestingly, out of the 549 AMR genes measured via Kruskal–Wallis, 289 genes had significant differences in relative abundance among body sites on farm A, and 271 genes differed on farm B. Of those, only 75 on farm A and 20 on farm B had higher gene prevalence in either rumen fluid or DNS samples than the fecal samples, confirming that AMR prevalence is generally higher in fecal samples.

### Metagenome Functional Annotation

The median number of unique functions identified by the *mi*-faser for each sampling site by farm is reported in Table 2. A greater number of unique functions were identified in fecal samples from farm B (*P* = 0.05). Still, no difference in functional annotation was detected in reads from rumen fluid or DNS samples between farms.

**TABLE 2:** Median number of unique functions identified per metagenome, by farm and sampling site [feces, rumen fluid, or nasopharyngeal swab (DNS)].

**Farm**	**Feces**		**Rumen**		**DNS**	
	**Median**	**IQR**	**Median**	**IQR**	**Median**	**IQR**
Farm A	765.50	152.25	693.50	101.50	109.50	127.50
Farm B	922.00	154.50	703.00	208.00	164.00	391.50

*IQR = interquartile range.*

**FIGURE 6:**
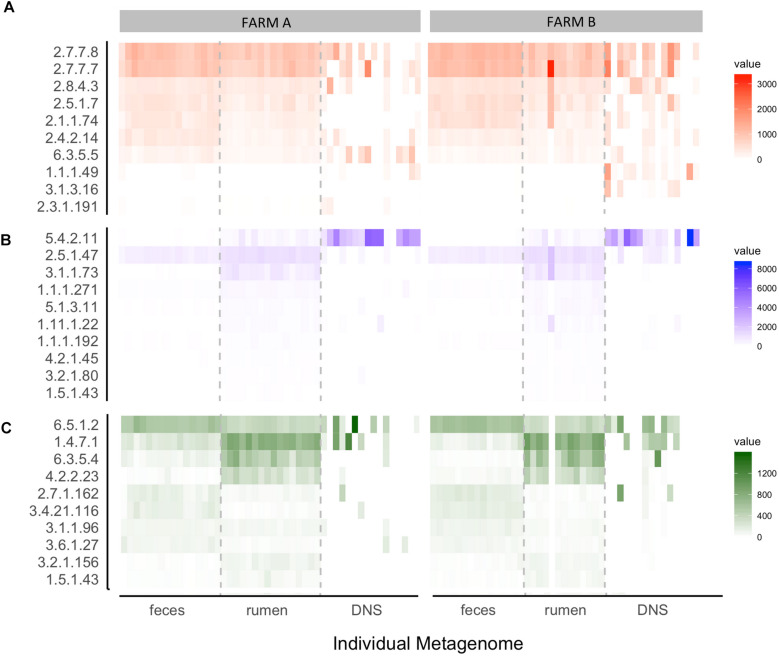
Heat map of the top 10 ECs selected by how well they distinguish **(A)** farm, **(B)** body site, and **(C)** farm given body site. Each box represents an individual sample and is shaded by its normalized read count value. Samples are stratified by farm **(A,B)** and body site from which the sample was taken [feces, rumen fluid, and deep nasal swab (DNS)]. ECs (Number: Class) = (A) 2.7.7.7: Nucleotidyltransferase, 2.7.7.8: Nucleotidyltransferase, 2.8.4.3: Methylsulfanyl transferase, 2.5.1.7: Alkyltransferase, 2.1.1.74: Methyltransferase, 2.4.2.14: Pentosyltransferase, 6.3.5.5: Carbon-nitrogen ligase, 1.1.1.49: Oxidoreductase, 3.1.3.16: Phosphoric - monoester hydrolase, 2.3.1.191: Acyltransferase. (B) 5.4.2.11: Phosphotransferase, 2.5.1.47: Alkyltransferase, 3.1.1.73: Carboxylic-ester hydrolase, 1.1.1.271: Oxidoreductase, 5.1.3.11: Epimerase, 1.1 1.1.22: Peroxidase, 1.1.1.192: Oxidoreductase, 4.2.1.45: Lyase, 3.2.1.80: Glycosylase, 1.5.1.43: Oxidoreductase. (C) 6.5.1.2: DNA ligase, 1.4.7.1: Oxidoreductase, 6.3.5.4: Carbon-Nitrogen Iigase, 4.2.2.23: Carbon-oxygen lyase, 2.7.1.162: Phosphotransferase, 3.4.21.1 16: Peptide hydrolase, 3.1.1.96: Carboxylic-ester hydrolase, 3.6.1.27: Nydr6lase, 3.2.1.156: Oiigosaccharide reducing-end xylanase, 1.5.1.43: Oxidoreductase.

Normalized read counts for the 10 highest ranked E.C.s for each nominal class were stratified by farm and sampling site and visualized on a heatmap (Figure 6). The normalized read counts for the 10 E.C.s most informative in predicting farm are given in Figure 6A, and those E.C.s were mapped to a number of KEGG pathways and biological functions, including microbial metabolism in diverse environments, lipopolysaccharide synthesis, and T cell receptor signaling (Table 3). The normalized read counts for the 10 E.C.s most informative in distinguishing body site are visualized in Figure 6B. Those E.C.s were mapped to a number of KEGG pathways and biological functions, including microbial metabolism in diverse environments, methane metabolism, and plant cell wall degradation (Table 4). The normalized read counts for the 10 E.C.s most informative in differentiating the composite farm by site nominal class are illustrated in Figure 6C, and those ECs were mapped to numerous KEGG pathways and biological functions, including microbial metabolism in diverse environments, amino acid metabolism, DNA repair, and gene expression checkpoints, as well as plant cell wall degradation (Table 5).

**TABLE 3:** Summary of informative E.C.s identified by the information gained with respect to “farm.”

**E.C.**	**Class**	**Pathways**	**Farm A**	**Farm B**
1.1.1.49	Oxidoreductase	Pentose phosphate; Glutathione metabolism; Metabolic pathways; Biosynthesis of secondary metabolites; Microbial metabolism in diverse environments	0	5.9
2.7.7.7	Nucleotidyltransferase	*DNA-directed DNA polymerase^1^*	797	1010
2.8.4.3	Methylsulfanyl transferase	*Involved in tRNA methylation^1^*	343.5	403
6.3.5.5	Carbon-nitrogen ligase	Pyrimidine metabolism; Alanine, aspartate, and glutamate metabolism; Metabolic pathways	167.9	123.2
2.5.1.7	Alkyltransferase	Amino sugar and nucleotide sugar metabolism; Peptidoglycan biosynthesis; Metabolic pathways	289.2	413.6
2.1.1.74	Methyltransferase	*Flavoprotein involved in post-translational modification^1^*	222.5	253
3.1.3.16	Phosphoric-monoester hydrolase	T cell receptor signaling; PD-L1 expression and PD-1 checkpoint pathway in cancer; Th1&Th2 cell differentiation	3.4	6.5
2.3.1.191	Acyltransferase	Lipopolysaccharide biosynthesis; Metabolic pathways	6.6	6.5
2.7.7.8	Nucleotidyltransferase	*Polyribonucleotide nucleotidyltransferase^1^*	867.5	1065.4
2.4.2.14	Pentosyltransferase	Purine metabolism; Alanine, aspartate and glutamate metabolism; Metabolic pathways; Biosynthesis of secondary metabolites	199.1	202.4

*Enzyme class, KEGG mapper implicated pathways, and the median normalized read count of each E.C. by farm is reported. ^1^General function, no specific pathways implicated.*

**TABLE 4:** Summary of informative E.C.s identified by the information gained with respect to “Site.”

**E.C.**	**Class**	**Pathways**	**Feces**	**Rumen**	**DNS^2^**
1.5.1.43	Oxidoreductase	Arginine and proline metabolism; Metabolic pathways	17.4	60.7	0
3.1.1.73	Carboxylic-ester hydrolase	*Helps break down plant cell wall hemicellulose^1^*	46.7	717.5	0
1.1.1.271	Oxidoreductase	Fructose and mannose metabolism; Amino sugar and nucleotide sugar metabolism; Metabolic pathways	104.9	265.2	0
5.4.2.11	Phosphotransferase	Glycolysis/Gluconeogenesis; Glycine, serine, and threonine metabolism; Methane metabolism; Metabolic pathways; Biosynthesis of secondary metabolites; Microbial metabolism in diverse environments	33.1	287.7	247109
5.1.3.11	Epimerase	*Catalyzes interconversion between D-glucose and D-mannose^1^*	45.5	190.6	0
1.1.1.192	Oxidoreductase	Fatty acid degradation	53.9	120.8	0
3.2.1.80	Glycosylase	Fructose and mannose metabolism	18.2	84.9	0
2.5.1.47	Alkyltransferase	Cysteine and methionine metabolism; Sulfur metabolism; Metabolic pathways; Biosynthesis of secondary metabolites; Microbial metabolism in diverse environments	652.1	1104.1	0
4.2.1.45	Lyase	Amino sugar and nucleotide sugar metabolism; Metabolic pathways	31.7	92.1	0
1.11.1.22	Peroxidase	*Protects cells from lipid peroxidation^1^*	15.8	180	0

*Enzyme class, KEGG mapper implicated pathways, and the median normalized read count of each E.C. by body site sampled is reported. ^1^General function, no specific pathways implicated. ^2^Deep nasal swab.*

**TABLE 5:** Summary of informative E.C.s identified by the information gained with respect to a compound “farm and Site” variable.

			**Farm A**			**Farm B**		
**E.C.**	**Class**	**Pathways**	**Feces**	**Rumen**	**DNS^2^**	**Feces**	**Rumen**	**DNS^2^**
1.4.7.1	Oxidoreductase	Glyoxylate and dicarboxylate metabolism; Nitrogen metabolism; Microbial metabolism in diverse environments	214.9	770.2	0	123.3	744.2	377.4
6.5.1.2	DNA ligase	*Forms phosphodiester bonds, repairing single-stranded DNA breaks^1^*	529.4	363.9	0	613.2	348.5	327.1
6.3.5.4	Carbon-Nitrogen ligase	Alanine, aspartate, and glutamate metabolism; Metabolic pathways, Biosynthesis of secondary metabolites	85.4	452.3	0	21.33	524.4	0
3.6.1.27	Hydrolase	Peptidoglycan biosynthesis	81.3	34.5	0	73.8	32.4	0
2.7.1.162	Phosphotransferase	*Lacto-N-biose/galacto-N-biose degradation in Bifidobacterium longum^1^*	145.3	51.4	0	178.1	24.8	0
4.2.2.23	Carbon-oxygen lyase	*Rhamnogalacturonan degradation in Bacillus subtilis and Aspergillus aculeatus^1^*	22.1	300.2	0	52.8	303.6	0
3.2.1.156	Oligosaccharide reducing-end xylanase	*Involved in breaking down plant cell wall hemicellulose^1^*	17.0	89.5	0	233.0	69.4	0
3.4.21.116	Peptide hydrolase	*Gene expression regulatory checkpoint; essential to formation of heat-resistant spores^1^*	135.7	23.9	0	160.6	23.4	0
1.5.1.43	Oxidoreductase	Arginine and proline metabolism; Metabolic pathways	18.0	56.1	0.	13.6	66.3	0
3.1.1.96	Carboxylic-ester hydrolase	*Cleaves mischarged tRNAAla; implicated in ethanol tolerance^1^*	89.4	71.4	0	100.5	54.7	0

*Enzyme class, KEGG mapper implicated pathways, and the median normalized read count of each E.C. by farm by Site is reported. ^1^General function, no specific pathways implicated. ^2^Deep nasal swab.*

## Discussion

To better understand the consequences of long-term exposure to contaminated drinking water in the metagenome of dairy cattle, we sequenced the metagenome of fecal, rumen fluid, and deep nasopharyngeal samples, and characterized the differences between animals in farms from heavy metal contaminated and non-contaminated areas. Our data revealed differences in bacterial taxa between cows from both areas, and the abundance and prevalence of AMR genes was higher in the affected farm (farm A) than the unaffected farm (farm B). This was the first study to assess the effect of an environmental in the metagenome of animals exposed to drinking water contaminated with high levels of heavy metals.

### Microbiome

Long-term exposure to heavy metals seemed to interfere in the fecal microbiota at phylum-level, since Firmicutes is described as the most abundant phylum in fecal samples of dairy cows in studies unrelated to heavy metal contamination (Liu et al., 2016; Xu et al., 2017; Hagey et al., 2019) and in the present research, Firmicutes was more abundant in farm B. Rodent models showed differences in gut microbiota after heavy metal exposure, possibly due to the direct effect of non-absorbed elements (Breton et al., 2013). In addition, the long-term effect of heavy metal pollution in the gut microbiota of *Bufo raddei* (Mongolian toad) revealed decreases in species diversity and in population of probiotic bacteria (Zhang et al., 2016). Our findings suggest that long-term exposure to heavy metal contaminated drink water may also interfere in the microbiota of dairy cattle feces at genus-level, probably due to the presence of more sensitive and more tolerant groups.

Analysis of rumen fluid at phylum-level revealed Bacteroidetes and Firmicutes as the major phyla. The rumen fluid microbiota is mainly composed of gram-negative bacteria in animals fed with high forage diets (Hungate, 1966). In our present research, dairy cattle from both herds had a forage-based diet and our results support this finding. *Fibrobacter succinogenes* (genus *Fibrobacter*) are butyrate-producing bacteria present in the rumen fluid, and this genus was higher in farm A compared to farm B. *Prevotella* and *Bacteroides* were the most abundant genera in almost all samples of this study. *Prevotella* has been reported to be the most abundant genus in the rumen (Chaucheyras-Durand and Ossa, 2014; Jami et al., 2014; Lima et al., 2015; Tong et al., 2018), whose species are related to degradation of starch, hemicellulose, and protein (Henderson et al., 2015). *Prevotella* has also been positively correlated with milk production (Lima et al., 2015; Indugu et al., 2017). Here, *Ruminococcus* and *Eubacterium* were numerically higher in farm A and farm B, respectively. The study conducted by Tong et al. (2018) showed higher abundances of *Ruminococcus 2* and *Eubacterium coprostanoligenes* in high yield cows. *Eubacterium* has also been associated with low residual feed intake (Elolimy et al., 2018), and this genus is involved in cellulose degradation in the rumen and some species are involved in the synthesis of butyrate (Flint et al., 2007), an energy source for cattle. We hypothesize that the lower abundance of *Eubacterium* and *Prevotella* in cows of farm A may be contributing to lower reported milk production for this farm. In fact, cows in farm A produced 250 L milk/per day before the dam failure, and, recently, the milk production is 200 L milk/per day.

Research on nasopharyngeal samples is often conducted to understand the bovine respiratory disease or changes in the microbiota in feedlot cattle. For the first time, we compared the deep nasopharyngeal microbiota of dairy cows from a potential heavy metal contaminated environment and a non-contaminated environment using animals with no signs of respiratory disease. Analysis at phylum-level revealed *Proteobacteria*, *Firmicutes*, *Actinobacteria*, and *Bacteroidetes* as the most abundant groups. Similar results were described by Lima et al. (2016) and Gaeta et al. (2016), in which studies *Proteobacteria* was also the most abundant phylum in healthy and pneumonic groups. At the genus-level, *Eubacterium*, *Streptococcus*, *Acinetobacter*, and *Bacillus* were the major groups observed in this study. These findings are in accordance with what has been previously reported, with *Acinetobacter* being commonly detected in the nasopharyngeal microbiota of cattle (Gaeta et al., 2016; Holman et al., 2017; Zeineldin et al., 2017). *Streptococcus* is an important genus related to pneumonia in humans, particularly *Streptococcus pneumoniae* (Jain et al., 2015), and has been detected in the respiratory tract of cattle (Klima et al., 2019). *Bacillus* are ubiquitously present in the environment and could be inhaled by cattle and detected in the upper respiratory tract. Some *Bacillus* species have the ability to produce antimicrobial peptides (Shelburne et al., 2006), that have been proven to inhibit the *in vitro* growth of *Pasteurella multocida*, *Mannheimia haemolytica*, and *Histophilus somni* (Xie et al., 2009), important pathogens involved in bovine respiratory disease complex. The presence of *Bacillus* as one of the major genus in the nasopharyngeal samples may be one reason for the lower abundance of *Pasteurella*, *Mannheimia*, and *Histophilus* observed in animals enrolled in this study, and, consequently, might have contributed to the absence of signs of respiratory disease.

### Resistome

Long-term persistence and continuous deposition of heavy metals in the environment (e.g., water source and soil) enable microbial interactions and transference of resistance genes, potentiating the emergence of AMR bacteria in food animals and increase the risk of human exposure through the food chain (primary production, food industry, and household) (Oniciuc et al., 2019; Pérez-Rodríguez and Mercanoglu Taban, 2019). Efflux pumps are protein systems related to cross-resistance (Baker-Austin et al., 2006; Martinez et al., 2009), as several antimicrobial molecules (e.g., heavy metals and antibiotics) are pumped out of the cell by the same system. Clinically important systems are members of RND superfamily in gram-negative bacteria (e.g., *Escherichia coli*, *Campylobacter jejuni*, and *Pseudomonas aeruginosa*), MFS and ABC in gram-positive bacteria and mycobacteria (e.g., *Staphylococcus aureus*, *Bacillus* spp., and *Mycobacterium tuberculosis*) (Piddock, 2006; Routh et al., 2011). Our results suggest that both cross-resistance and co-resistance mechanisms may be occurring more often in farm A due to the higher prevalence of efflux pumps (including multi-metal resistance RND and multi-biocide resistance ABC), in addition to protein types conferring resistance to several antibiotic classes, possibly enhanced by environmental contamination. Furthermore, the detection of several resistance mechanisms warn for possible presence of multidrug resistant bacteria in feces and rumen that may contaminate raw milk (Tóth et al., 2020), carcasses, abattoir surfaces, workers, and consumers, which have been extensively documented in the literature (Schlegelová et al., 2004; Lavilla Lerma et al., 2014; Savin et al., 2020).

AMR development is a natural event that can be exacerbated by therapy administration and/or growth promoters (Pérez-Rodríguez and Mercanoglu Taban, 2019). In the present research, we evaluate the long-term exposure to heavy-metal contaminated drinking water in dairy cows as a potential new pathway to AMR development in farm animals. Our data showed that fecal samples exhibited the highest prevalence of AMR genes compared to rumen fluid and DNS, which implicates future sampling designs using shotgun sequencing.

### Functional Annotation

The symbiotic relationships among rumen fluid microbial communities are responsible for producing microbial protein by ammonia nitrogen utilization. The ammonia assimilation occurs by two main pathways in which the participation of glutamate synthase is fundamental. This enzyme is classified according to the electron donor; NADH-dependent, NADPH-dependent, which is commonly found in several bacteria, and the ferredoxin-dependent glutamate synthase, which is found in plants and cyanobacteria (Pengpeng and Tan, 2013). Here, the results showed that ferredoxin-dependent glutamate synthase (E.C. 1.4.7.1) was overrepresented in rumen fluid samples of both farms, and in DNS of farm B. *Cyanobacteria* were overrepresented in two DNS samples of farm B, which may explain the results for this site. Glutamine-dependent asparagine synthase (E.C. 6.3.5.4), also identified in ammonia assimilation in rumen bacteria (Pengpeng and Tan, 2013), was overrepresented on farm B ruminal samples. This in addition to the presence of ferredoxin-dependent glutamate synthase suggests that the production of microbial protein is higher in the rumen environment of farm B dairy cows.

The 2,3-diphosphoglycerate dependent phosphoglycerate mutase (E.C. 5.4.2.11) was overrepresented in rumen fluid compared to feces but highly overrepresented in DNS, and probably independent of farm. A metal independent enzyme is present in vertebrates, yeast, and several bacteria (e.g., *Haemophilus influenzae*). This enzyme participates in glycolysis and gluconeogenesis processes and methane metabolism (Jedrzejas, 2000). In addition, DNA ligase (E.C. 6.5.1.2) is also represented in feces and rumen fluid, but it is overrepresented in DNS samples of farm B. This enzyme, commonly found in bacteria, participates in the DNA replication by forming phosphoric-ester bonds and repairs DNA breaks. The NADP-dependent glucose 6-phosphate dehydrogenase (E.C. 1.1.1.49), overrepresented in farm B, participates in the pentose phosphate pathway, which mainly produces NADPH and ribose-5-phosphate, a precursor of nucleic acids (Stanton, 2012). Finally, the DNA-directed DNA polymerase (E.C. 2.7.7.7), also overrepresented in farm B, is responsible for the synthesis of a new strand of DNA by adding nucleotides (Roettger et al., 2010), and it is also implicated in repair of DNA breaks. Taken together, these findings lead us to hypothesize that cows of farm B have more active bacterial replication compared to farm A, and bacteria in farm A are more susceptible to mutation and death, as they might have decreased ability to maintain DNA integrity. Thus, more research is required to understand differences in bacterial metabolism and behavior in cows of heavy metal contaminated and non-contaminated farms. It is worth mentioning that DNS samples were characterized by high variation in functional annotation, which may be due to low quantity DNA and the difficulty of DNA extraction from swabs. To better understand what is occurring in the nasopharyngeal environment, sampling and extraction protocols need to be optimized with more representative samples.

This broad, exploratory study is limited in the ability to draw mechanistic conclusions. One notable limitation is the lack of baseline pre-environmental disaster data to identify if the metagenome and resistome differed between the two farms pre-exposure. Because the AMR gene count data was normalized and scaled to account for gene length and sequencing depth bias, count-based statistical analyses (i.e., alpha and beta diversity, differential abundance analysis) were not possible. Also, diet can influence microbiota of dairy cattle (Loor et al., 2016; Dill-McFarland et al., 2018) but we did not directly measure this effect. In addition, cows within each farm were given the same diet, and the confounding effects of diet are already accounted for by a farm comparison. All samples were processed at once in the same batch, eliminating concerns regarding a true batch effect in terms of contamination or sequencing errors. Cows were the same breed, though we do not have information regarding their relatedness.

## Conclusion

Long-term persistence of heavy metals in the environment may interfere in the microbiome of dairy cows. These data suggest that exposure to heavy metal contamination results in selection for bacteria that confer resistance to biocides, drugs, and metals and that AMR genes are most readily detected in fecal samples compared to the rumen fluid and nasopharyngeal samples. In addition, metagenome functional annotation data suggested that selective pressures of heavy metal exposure could be skewing pathway diversity toward fewer, more specialized, functions. Differences in microbiota indicated that exposure to heavy metal contaminated water may interfere in dairy cattle microbiota and, consequently, in animal productivity. Further research is warranted to determine if AMR is transferred via the food chain by milk or meat consumption, which would have substantial implications for human health. Since heavy metal contamination has an effect on animal microbiomes, environmental management, such as correct dam construction and maintenance and AMR surveys are warranted to protect the food system from hazardous consequences.

## Data Availability Statement

The datasets presented in this study can be found in online repositories. The name of the repository/repositories can be found below: https://www.mg-rast.org/linkin.cgi?project=mgp92419, https://github.com/gandalab/amr-heavymetal.

## Ethics Statement

The animal study was reviewed and approved by Ethics Committee in the Use of Animals of the School of Veterinary Medicine and Animal Science of the University of São Paulo (protocol no. 6424070217). Written informed consent was obtained from the owners for the participation of their animals in this study.

## Author Contributions

NG, LG, and EG contributed to conception and design of the study. NG, DC, JC, and MA collected the data. NG, EB, and AM organized the database. NG, EB, and AM performed the statistical analysis. NG wrote the first draft of the manuscript. EB and AM wrote sections of the manuscript. All authors contributed to manuscript revision, read, and approved the submitted version.

## Conflict of Interest

The authors declare that the research was conducted in the absence of any commercial or financial relationships that could be construed as a potential conflict of interest.

## Footnotes

1 https://megares.meglab.org/download/index.php

2 https://services.bromberglab.org/mifaser/submit

## Supplementary Material

Supplementary Figure S1 Distribution of most prevalent phyla across samples. Percentage relative abundance of the most prevalent bacterial phyla identified in **(A)** fecal swab, **(B)** rumen fluid and **(C)** deep nasopharyngeal swab of dairy cattle and for farm A (left) and farm B (right). *X* axis represent samples: numbers represent each cow; A represents affected farm and B the unaffected farm.

Supplementary Figure S2 Percentage relative abundance of the most prevalent bacterial genera identified in **(A)** fecal swab, **(B)** rumen fluid and **(C)** deep nasopharyngeal swab of dairy cattle and for farm A (left) and farm B (right). *X* axis represent samples: numbers represent each cow; A represents affected farm and B the unaffected farm.

Supplementary Figure S3 AMR gene distribution across samples is highly heterogeneous and over 50% of AMR genes are present in fewer than 10% of samples. Antimicrobial resistance gene presence is heterogeneous across samples. The number of genes in each range is shown at the top of the bar. Over 50% of AMR genes (317 of 549) are present in fewer than 10% of samples. One gene (PH23S for Phenicol resistance) is present in all samples. The one gene present in all samples: Drugs| Phenicol| Phenicol-resistant_23S_rRNA_mutation| PH23S| RequiresSNPConfirmation.

Supplementary Figure S4 Drug resistance prevalence and sample heterogeneity across farms. Drug resistance gene presence across farms. The top (blue) bar plot and dot matrix are the number of samples that share common gene presence. The left (gray) histogram is the frequency of each drug resistance type across samples in each.

Supplementary Table S1 DNS genera that had the most differential abundance (largest log2 fold change) between farm A and farm B. The 25 most increased genera in farm A and the 25 most increased genera in farm B are shown. *P* values were adjusted with False Discovery Rate.

Supplementary Table S2 Ruminal fluid genera that had the most differential abundance (largest log2 fold change) between farm A and farm B. The 25 most increased genera in farm A and the 25 most increased genera in farm B are shown. *P* values were adjusted with False Discovery Rate.

Supplementary Table S3 DNS genera that had the most differential abundance (largest log2 fold change) between farm A and farm B. The 25 most increased genera in farm A and the 25 most increased genera in farm B are shown. *P* values were adjusted with False Discovery Rate.
