# Supplementary material for: A Cross-Sectional Study of Dairy Cattle Metagenomes Reveals Increased Antimicrobial Resistance in Animals Farmed in a Heavy Metal Contaminated Environment
Source: Front Microbiol. 2020 Nov 16;11:590325. doi: 10.3389/fmicb.2020.590325 (PMC7701293; doi:10.3389/fmicb.2020.590325)
Supplement: Supplementary Figure S1 — Distribution of most prevalent phyla across samples. Percentage relative abundance of the most prevalent bacterial phyla identified in (A) fecal swab, (B) rumen fluid and (C) deep nasopharyngeal swab of dairy cattle and for farm A (left) and farm B (right). X axis represent samples: numbers represent each cow; A represents affected farm and B the unaffected farm. [file Data_Sheet_1.docx]

Supplementary Material


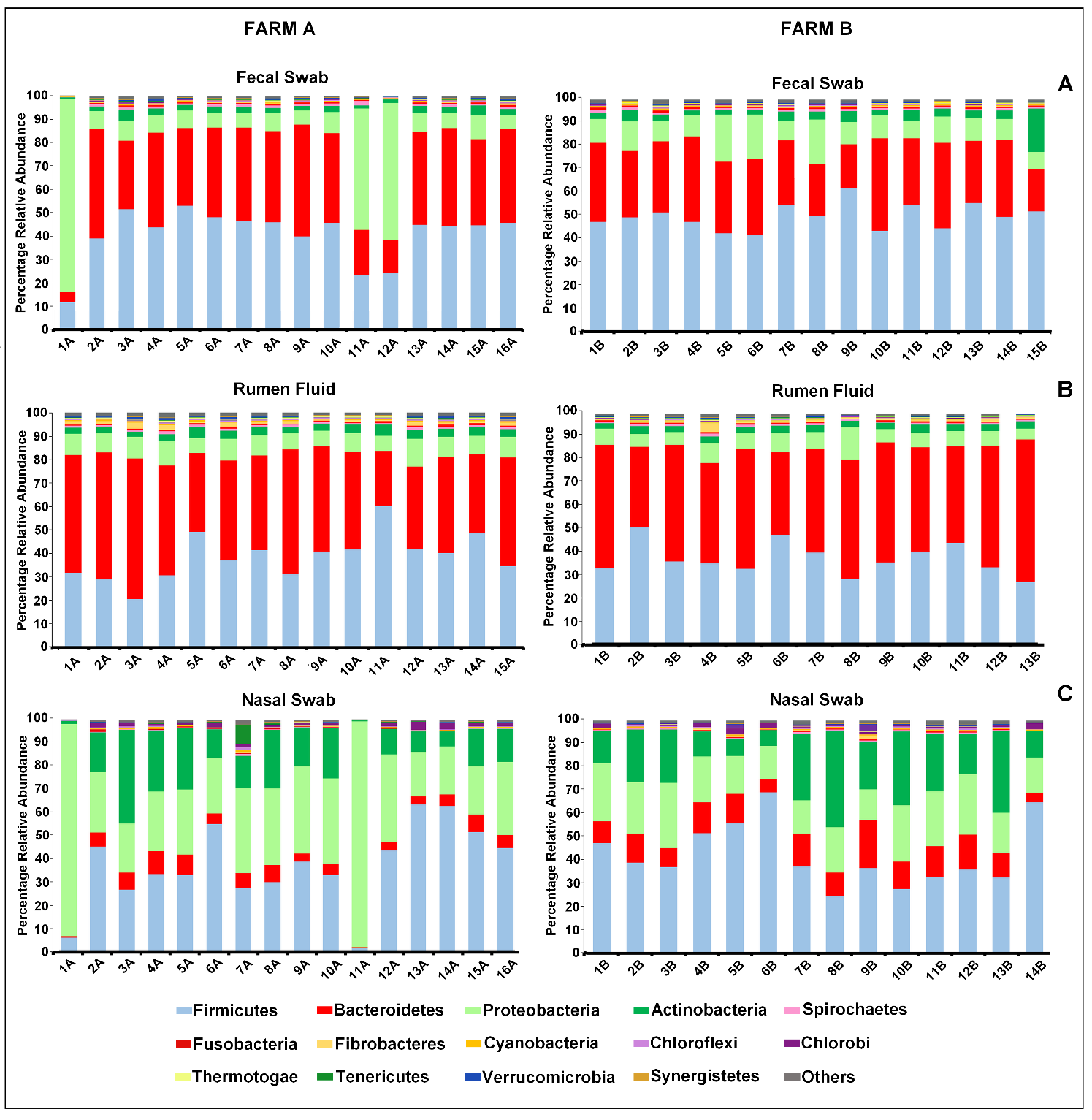


**Supplementary Figure 1. Distribution of most prevalent phyla across samples.** Percentage relative abundance of the most prevalent bacterial phyla identified in (A) fecal swab, (B) rumen fluid and (C) deep nasopharyngeal swab of dairy cattle and for farm A (left) and farm B (right). X axis represents samples; numbers represent each cow; A represents affected farm and B the unaffected farm.

**
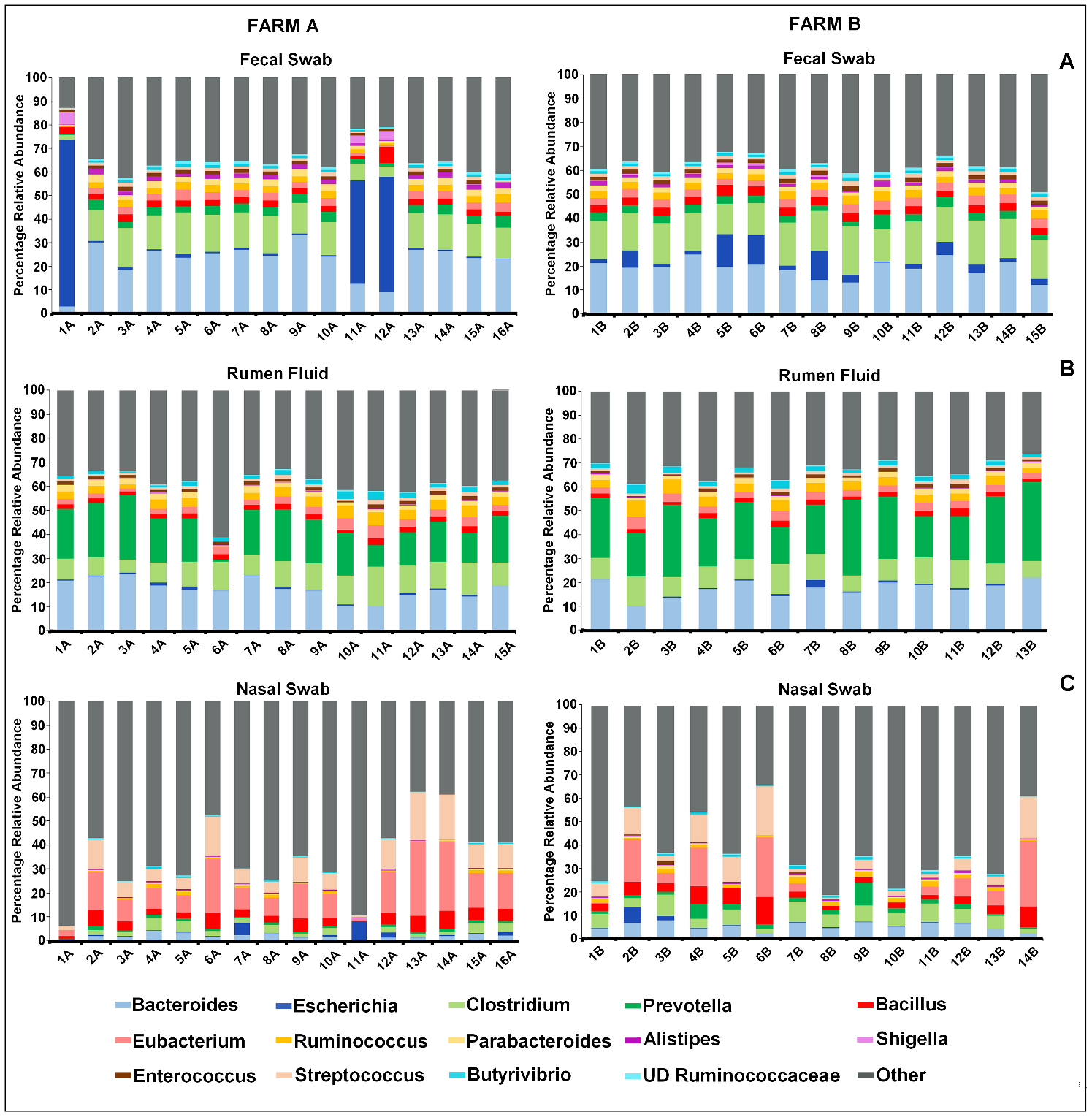
**

**Supplementary Figure 2.** Percentage relative abundance of the most prevalent bacterial genera identified in (A) fecal swab, (B) rumen fluid and (C) deep nasopharyngeal swab of dairy cattle and for farm A (left) and farm B (right). X axis represent samples: numbers represent each cow; A represents affected farm and B the unaffected farm. UD Ruminococcaceae means Undetermined (from Ruminococcaceae)

**
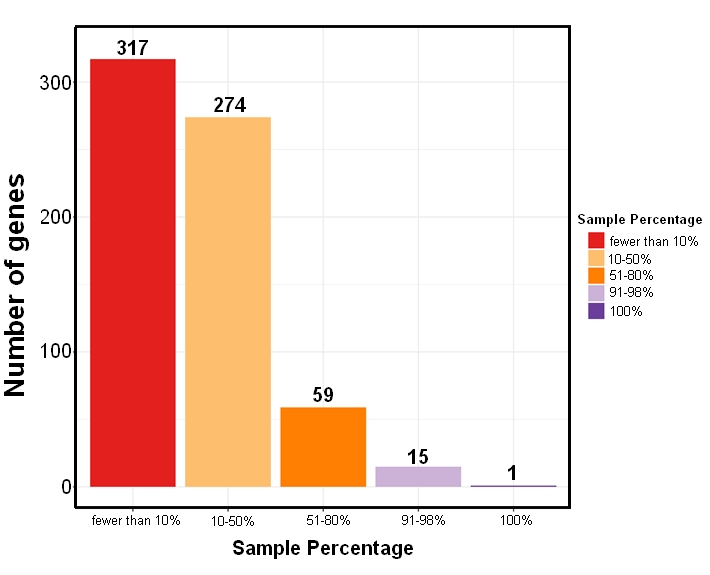
**

**Supplementary Figure 3.** **AMR gene distribution across samples is highly heterogeneous and over 50% of AMR genes are present in fewer than 10% of samples.** Antimicrobial resistance gene presence is heterogeneous across samples. The number of genes in each range is shown at the top of the bar. Over 50% of AMR genes (317 of 549 are present in fewer than 10% of samples. One gene (PH23S for Phenicol resistance) is present in all samples. The one gene present in all samples: Drugs|Phenicol|Phenicol-resistant_23S_rRNA_mutation|PH23S|RequiresSNPConfirmation

**
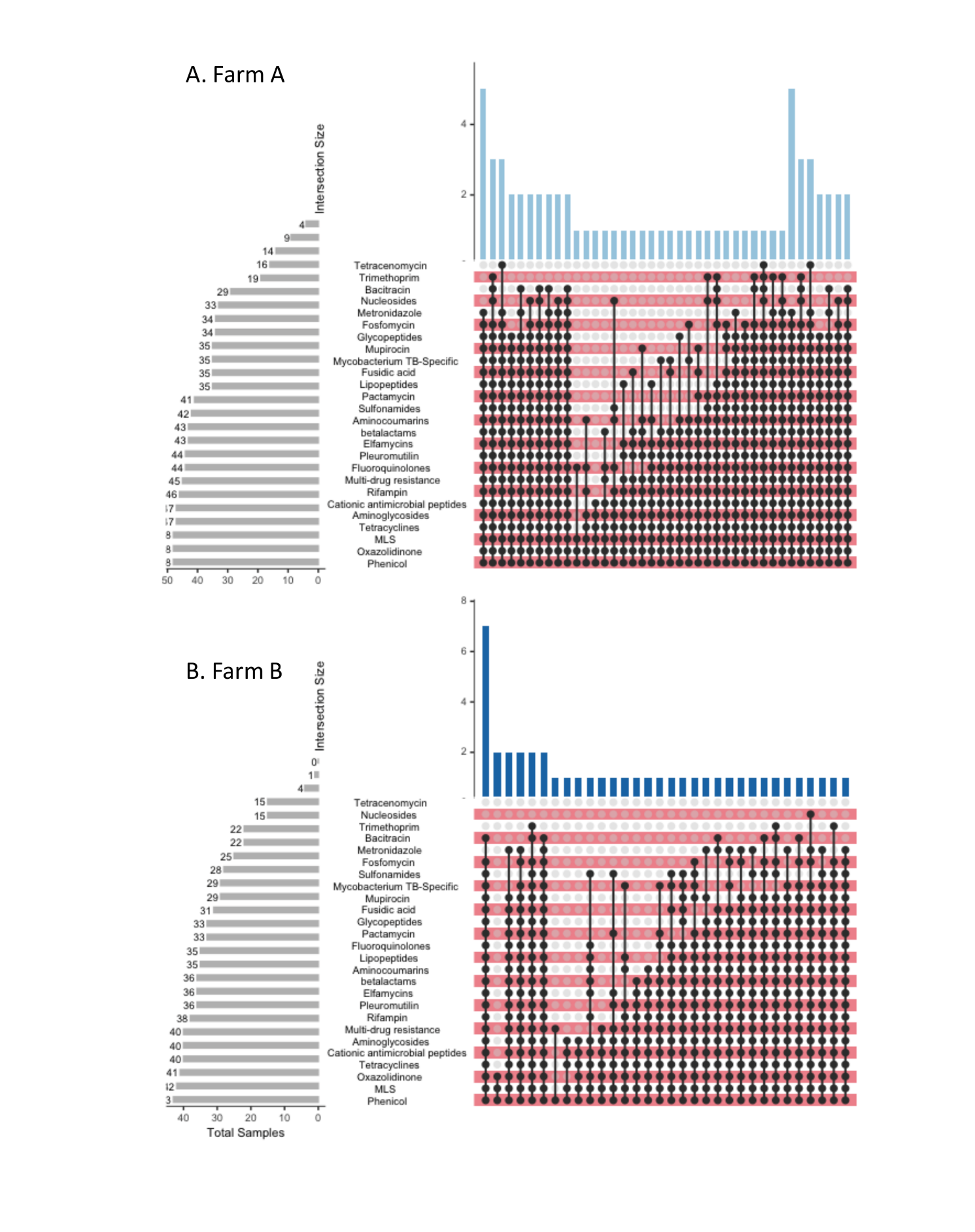
**

**Supplementary Figure 4.** **Drug resistance prevalence and sample heterogeneity across farms.** Drug resistance gene presence across farms. The top (blue) bar plot and dot matrix are the number of samples that share common gene presence. The left (grey) histogram is the frequency of each drug resistance type across samples in each

**Supplementary Table 1** Fecal genera that had the most differential abundance (largest log2 fold change) between farm A and farm B. The 25 most increased genera in farm A and the 25 most increased genera in farm B are shown. P values were adjusted with False Discovery Rate.

| Genus | Mean relative abundance | Log2 fold change | Adjusted *P* value |
| --- | --- | --- | --- |
| *Bdellovibrio* | 910.304 | -29.787 | 0.000 |
| *Bifidobacterium* | 366718.602 | -2.438 | 0.000 |
| *Simonsiella* | 7346.283 | -1.213 | 0.043 |
| *Kingella* | 6015.586 | -1.152 | 0.045 |
| *Candidatus.Carsonella* | 23.909 | -1.009 | 0.003 |
| *Lysinibacillus* | 18889.783 | -0.562 | 0.025 |
| *Gardnerella* | 12801.089 | -0.556 | 0.012 |
| *Bulleidia* | 12577.781 | -0.516 | 0.007 |
| *Pseudoramibacter* | 15133.424 | -0.486 | 0.001 |
| *Parascardovia* | 3821.974 | -0.474 | 0.023 |
| *Olsenella* | 26089.110 | -0.418 | 0.033 |
| *Mesoplasma* | 3140.035 | -0.400 | 0.002 |
| *Fibrobacter* | 53889.196 | -0.365 | 0.003 |
| *Scardovia* | 2425.511 | -0.336 | 0.029 |
| *Candidatus Phytoplasma* | 6247.347 | -0.335 | 0.000 |
| *Bacillus* | 771183.869 | -0.329 | 0.033 |
| *Mycoplasma* | 23331.924 | -0.310 | 0.001 |
| *Tropheryma* | 955.438 | -0.306 | 0.026 |
| *Erysipelothrix* | 7548.085 | -0.289 | 0.003 |
| *Catenibacterium* | 29388.004 | -0.258 | 0.045 |
| *Unclassified^1^* | 8298.187 | -0.253 | 0.000 |
| *Acholeplasma* | 23530.319 | -0.252 | 0.004 |
| *Segniliparus* | 641.131 | -0.251 | 0.030 |
| *Raphidiopsis* | 434.913 | -0.249 | 0.023 |
| *Anaerofustis* | 24876.192 | -0.246 | 0.000 |
| *Unclassified^2^* | 1249.010 | 0.521 | 0.014 |
| *Dickeya* | 4803.101 | 0.525 | 0.005 |
| *Pasteurella* | 5817.469 | 0.533 | 0.003 |
| *Pectobacterium* | 7042.003 | 0.538 | 0.008 |
| *Alicycliphilus* | 835.448 | 0.542 | 0.029 |
| *Burkholderia* | 61921.764 | 0.556 | 0.045 |
| *Janthinobacterium* | 3183.634 | 0.557 | 0.015 |
| *Thiomonas* | 1128.572 | 0.565 | 0.023 |
| *Alistipes* | 426581.018 | 0.573 | 0.001 |
| *Serratia* | 6820.960 | 0.593 | 0.007 |
| *Edwardsiella* | 3689.614 | 0.595 | 0.003 |
| *Pseudomonas* | 54866.676 | 0.614 | 0.004 |
| *Azotobacter* | 2114.930 | 0.615 | 0.006 |
| *Cupriavidus* | 9328.752 | 0.616 | 0.010 |
| *Ralstonia* | 8291.862 | 0.639 | 0.017 |
| *Moritella* | 1603.244 | 0.645 | 0.008 |
| *Aeromonas* | 12909.663 | 0.674 | 0.005 |
| *Treponema* | 134253.906 | 0.686 | 0.035 |
| *Unclassified^3^* | 1084.089 | 0.692 | 0.003 |
| *Erwinia* | 3831.015 | 0.695 | 0.010 |
| *Tolumonas* | 6707.355 | 0.732 | 0.016 |
| *Herbaspirillum* | 1862.015 | 0.767 | 0.005 |
| *Limnobacter* | 1055.594 | 0.863 | 0.010 |
| *Unclassified^4^* | 24.576 | 0.912 | 0.034 |
| *Corynebacterium* | 73049.239 | 0.963 | 0.036 |

1 derived from Clostridiales family

2 derived from Vibrionaceae

3 derived from Alteromonadales

4 derived from Proteobacteria

**Supplementary Table 2.** Ruminal fluid genera that had the most differential abundance (largest log2 fold change) between farm A and farm B. The 25 most increased genera in farm A and the 25 most increased genera in farm B are shown. P values were adjusted with False Discovery Rate.

| Genus | Mean relative abundance | Log2 fold change | Adjusted *P* value |
| --- | --- | --- | --- |
| *Fulvimarina* | 5208.396 | -5.342 | 0.000 |
| *Gordonia* | 2550.295 | -3.609 | 0.000 |
| *Avibacterium* | 0.106 | -3.089 | 0.008 |
| *Gemmatimonas* | 4604.636 | -3.026 | 0.000 |
| *Collimonas* | 0.612 | -1.898 | 0.026 |
| *Unclassified^1^* | 1.842 | -1.886 | 0.001 |
| *Intrasporangium* | 1084.934 | -1.829 | 0.025 |
| *Acetobacter* | 2605.809 | -1.658 | 0.005 |
| *Glaciecola* | 383.684 | -1.599 | 0.012 |
| *Acinetobacter* | 20519.791 | -1.589 | 0.000 |
| *Gallionella* | 1189.287 | -1.570 | 0.004 |
| *Mitsuokella* | 17015.582 | -1.253 | 0.011 |
| *Spiroplasma* | 8.662 | -1.192 | 0.001 |
| *Moritella* | 911.120 | -1.161 | 0.026 |
| *Unclassified^2^* | 2254.545 | -1.160 | 0.006 |
| *Bifidobacterium* | 68219.712 | -1.054 | 0.001 |
| *Candidatus.Regiella* | 166.465 | -0.997 | 0.028 |
| *Comamonas* | 970.074 | -0.887 | 0.001 |
| *Catenibacterium* | 12735.211 | -0.858 | 0.020 |
| *Mannheimia* | 2577.847 | -0.824 | 0.015 |
| *Xenorhabdus* | 964.483 | -0.813 | 0.025 |
| *Psychrobacter* | 2904.251 | -0.694 | 0.043 |
| *Histophilus* | 2747.568 | -0.679 | 0.007 |
| *Acidovorax* | 4271.517 | -0.663 | 0.025 |
| *Moraxella* | 585.789 | -0.658 | 0.020 |
| *Helicobacter* | 10609.854 | -0.625 | 0.037 |
| *Deinococcus* | 3720.643 | 0.180 | 0.049 |
| *Chloroflexus* | 5530.668 | 0.183 | 0.026 |
| *Hyphomonas* | 994.567 | 0.211 | 0.024 |
| *Thermus* | 2476.404 | 0.243 | 0.001 |
| *Desulfobacterium* | 2203.256 | 0.264 | 0.025 |
| *Desulfomicrobium* | 2164.776 | 0.269 | 0.037 |
| *Acidobacterium* | 2189.662 | 0.275 | 0.037 |
| *Ketogulonicigenium* | 263.261 | 0.286 | 0.004 |
| *Nitrospira* | 815.240 | 0.287 | 0.006 |
| *Desulfurivibrio* | 1419.303 | 0.305 | 0.029 |
| *Oscillochloris* | 537.337 | 0.311 | 0.020 |
| *Desulfatibacillum* | 3490.506 | 0.321 | 0.020 |
| *Desulfuromonas* | 2231.851 | 0.323 | 0.016 |
| *Desulfohalobium* | 1234.347 | 0.326 | 0.025 |
| *Hoeflea* | 235.520 | 0.334 | 0.020 |
| *Acidimicrobium* | 277.185 | 0.334 | 0.008 |
| *Desulfonatronospira* | 803.258 | 0.352 | 0.026 |
| *Unclassified^3^* | 2232.897 | 0.359 | 0.038 |
| *Blastopirellula* | 2505.721 | 0.394 | 0.025 |
| *Cyanobium* | 173.489 | 0.401 | 0.007 |
| *Elusimicrobium* | 4304.043 | 0.725 | 0.020 |
| *Methylacidiphilum* | 1255.777 | 0.829 | 0.012 |
| *Chthoniobacter* | 2374.111 | 0.852 | 0.025 |
| *Victivallis* | 15892.275 | 1.245 | 0.003 |

1 derived from Thiotrichales

2 derived from Burkholderiales

3 derived from Deltaproteobacteria

**Supplementary Table 3.** DNS genera that had the most differential abundance (largest log2 fold change) between farm A and farm B. The 25 most increased genera in farm A and the 25 most increased genera in farm B are shown. P values were adjusted with False Discovery Rate.

| Genus | Mean relative abundance | Log2 fold change | Adjusted *P* value |
| --- | --- | --- | --- |
| *Fluoribacter* | 29.150 | -7.777 | 0.000 |
| *Coraliomargarita* | 107.147 | -6.037 | 0.000 |
| *Nautilia* | 41.133 | -5.963 | 0.000 |
| *Collimonas* | 8.857 | -5.554 | 0.002 |
| *Cylindrospermopsis* | 13.845 | -5.177 | 0.000 |
| *Parachlamydia* | 20.180 | -5.105 | 0.000 |
| *Ethanoligenens* | 620.060 | -4.757 | 0.000 |
| *Fervidobacterium* | 43.296 | -4.702 | 0.000 |
| *Mesoplasma* | 10.428 | -4.633 | 0.000 |
| *Kosmotoga* | 14.967 | -4.536 | 0.000 |
| *Oxalobacter* | 60.586 | -4.506 | 0.000 |
| *Marinococcus* | 2.730 | -4.349 | 0.004 |
| *Leptospirillum* | 2.052 | -4.232 | 0.010 |
| *Mucilaginibacter* | 80.864 | -4.016 | 0.000 |
| *Ornithobacterium* | 1.278 | -3.653 | 0.000 |
| *Nitratiruptor* | 12.404 | -3.442 | 0.000 |
| *Fulvimarina* | 33.524 | -3.361 | 0.000 |
| *Limnobacter* | 20.094 | -3.322 | 0.000 |
| *Persephonella* | 5.256 | -3.281 | 0.000 |
| *Hoeflea* | 29.752 | -3.248 | 0.000 |
| *Lyngbya* | 13.683 | -3.202 | 0.000 |
| *Gemmatimonas* | 47.276 | -3.182 | 0.000 |
| *Methylovorus* | 26.102 | -3.041 | 0.000 |
| *Dokdonia* | 40.232 | -3.037 | 0.000 |
| *Pediococcus* | 52.814 | -2.924 | 0.000 |
| *Ochrobactrum* | 23.108 | 1.972 | 0.000 |
| *Klebsiella* | 101.864 | 1.992 | 0.015 |
| *Methylobacterium* | 97.841 | 2.093 | 0.000 |
| *Orientia* | 0.586 | 2.150 | 0.018 |
| *Enterobacter* | 97.650 | 2.325 | 0.004 |
| *Nitrobacter* | 59.965 | 2.364 | 0.000 |
| *Escherichia* | 246.200 | 2.451 | 0.000 |
| *Fusobacterium* | 42.305 | 2.479 | 0.000 |
| *Unclassified* | 61.007 | 2.511 | 0.000 |
| *Pectobacterium* | 62.186 | 2.609 | 0.002 |
| *Parvibaculum* | 12.491 | 2.643 | 0.000 |
| *Yersinia* | 95.040 | 2.644 | 0.000 |
| *Cupriavidus* | 91.512 | 2.668 | 0.000 |
| *Shigella* | 32.558 | 2.688 | 0.000 |
| *Salmonella* | 77.891 | 2.749 | 0.000 |
| *Citromicrobium* | 47.885 | 2.802 | 0.000 |
| *Sorangium* | 14.979 | 2.889 | 0.002 |
| *Geodermatophilus* | 26.220 | 3.069 | 0.000 |
| *Paracoccus* | 53.542 | 3.359 | 0.000 |
| *Serratia* | 93.636 | 3.370 | 0.000 |
| *Cronobacter* | 66.221 | 3.416 | 0.000 |
| *Pedobacter* | 30.946 | 4.078 | 0.000 |
| *Erwinia* | 64.472 | 4.459 | 0.000 |
| *Parabacteroides* | 44.019 | 4.841 | 0.000 |
| *Planctomyces* | 7.168 | 6.003 | 0.000 |

*Derived from Rickettsiales
